# Attitudes of Local Population Towards the Impacts of Tourism Development: Evidence From Czechia

**DOI:** 10.3389/fpsyg.2021.684773

**Published:** 2021-06-01

**Authors:** Ivica Linderová, Petr Scholz, Nuno Almeida

**Affiliations:** ^1^Department of Travel and Tourism, College of Polytechnics Jihlava, Jihlava, Czechia; ^2^CiTUR, School of Tourism and Maritime Technology, Polytechnic of Leiria, Peniche, Portugal

**Keywords:** tourism development, locals, Doxey irritation index, tourism area life cycle, rural area, Czechia

## Abstract

Increasing the socio-economic effects caused by the tourism development in the local population, they adopt some attitudes according to the impacts directly or indirectly perceived. However, some of this impact can be considered positive or negative, according to different perspectives. The issue of the resident-tourist relationship has been much-discussed recently. Therefore, many case studies are being conducted that address the impacts on both residents and tourists. The goal of this manuscript is to analyze the attitudes of local residents to the development of tourism in the urban monument zone Předhradí. Primary data were collected in a questionnaire survey for residents who have a permanent residence in a municipality of Předhradí in 2020. In our research, we tried to identify the significant negative impacts of tourism development. In the same way, we evaluate how the locals see positive effects on their quality of life conducted with tourism development. The research finds out that local respondents perceived some negative impacts to increase the economic perspective, as they referred to in the higher traffic load or increased noise. The pandemic crises are perceived as a game-changer in the tourism industry. For that reason, we suggest the primary considerations for future research not only with the academic perfective as for the practical point of view. The local population’s entrepreneurship attitudes must be one of the tools to assume the resilience toward the tourism development impacts.

## Introduction

Tourism has played in history and may play in the future a positive and significant role in society, although it can also bring adverse socio-economic effects if managed poorly (Marković and Klarić, 2015). The tourism industry holds a significant economic significance and is continually and dynamically developing (Linderová and Janeček, 2017). Tourism, the third-largest sector in the world, represents 10% of the world’s GDP, and it is also responsible for 5% of the world’s carbon dioxide emissions (Migale et al., 2019). As tourism evolved, its original meaning of traveling to the unknown, to places outside of the ordinary, where visitors experience a sense of excitement by directly contacting the local community, has changed (Pompurová et al., 2020). We can state that the possible negative socio-economic effects certainly stand out from the local identity’s weakening under tourism and weekend visitors’ influence (Marković and Klarić, 2015). The issue of the resident-tourist relationship has been much-discussed recently. Therefore, many case studies are being conducted that address the impacts on both residents and tourists. Great emphasis needs to be placed on maintaining good relations between these groups, as this is the cornerstone of sustainable tourism. Marković and Klarić (2015) further state that the next potential threat is the consumption of the most valuable areas for tourism, thereby reducing the possibility of using it by the domestic population.

A rural area is an open swath of land with few homes or other buildings and not very many people. A rural areas population density is very low. Many people live in a city, or urban area. Their homes and businesses are located very close to one another (Stanley, 2011). Sociologists commonly describe the ideal type of personal contacts in cities and towns as anonymous, segmental, and impersonal and contrast them with the intimate and personal type in rural areas (Simmel, 1971). In a rural area, there are fewer people, and their homes and businesses are located far away from one another, e.g., in the United States, rural areas take up about 98% of the country but are home to only 25% of the population. In Ethiopia, a less-developed country where agricultural jobs are much more common, 87% of the population live in rural areas (Stanley, 2011).

In numerous countries, rural areas are less developed areas that have many specific problems. A typical example is Czechia, where rural areas have been out of the general public interest for many years. It resulted in an undesired situation of Czech farmers (particularly the small ones), a high unemployment rate, shortage of finance and legislative support, which would promote business investments and new job creation. The situation has changed after the European Union’s (EU) entry. Generally, the EU member states want to increase the quality of life, clear or mitigate regional disparity and keep sustainable development in rural areas (Šimková, 2007). According to the UNWTO report, tourism movements will increase annually by 3–5% in the next few years. Tourist expectations will also change in conjunction with this increase. These visitors, also called new tourists, prefer holidays oriented to their interest and buy tourism products that are more based on nature, authenticity, and experience. Rural tourism, as one of the new types of tourism, has been increasingly prevalent in the tourism industry (Ayazlar and Ayazlar, 2015).

Rural areas are intrinsically essential and fundamentally different from urban areas and thus (often) require a different set of interventions and policies that aim to improve their populations’ livelihood. Research and empirical evidence show that rural areas are characterized by slow dynamics of farm productivity, widespread income inequality, and volatility of agricultural income; considerable outward migration flows to urban areas that result in the depopulation of rural areas; a lack of efficient physical, technological and information technology infrastructures; public and private services that are more costly to provide and more difficult to access than in urban areas (OECD, 2020).

Despite their importance, rural statistics on income and livelihoods are sparse and uncommon, mainly since there is no consistent international definition of rural areas. Rural areas are usually defined based on national policy objectives; sometimes, as a residual, once urban areas are defined, or periodically based on a combination of multiple criteria, for example, population size and density, the presence of agriculture, remoteness from urban areas and a lack of infrastructure and/or basic social services (Dijkstra et al., 2021).

The area considered rural is defined concerning the areas deemed to be urban. Often what is rural is simply what is not urban. Still, it is easy to think of different kinds or degrees of rurality, from fairly populated areas with extensive cropland to less densely settled areas of sparse vegetation. Rural definitions most usually address one or more of three dimensions that characterize differences between urban and rural areas and among rural areas (Food and Agriculture Organization of the United Nations (FAO), 2018): (1) The sparse settlement reflects the idea that urban areas are those that have the most people and that most densely settled, while rural areas are more sparsely populated and settled. (2) Land cover is the physical cover on the land, including vegetation (either planted or naturally occurring) and any buildings or features constructed by humans. The land cover follows and determines land use related to the human activities that occur there. (3) Remoteness affects the opportunities people have to gain access to markets and public services. It is most often represented by the difficulty of physical travel to places where markets and services are more available.

Urban-rural typology statistics use the new urban-rural typology. This typology uses a three-step approach to classify the NUTS (Nomenclature of Territorial Units for Statistics) level three regions. Rural areas are all areas outside urban clusters. Urban clusters are clusters of contiguous grid cells of 1 km^2^ with a density of at least 300 inhabitants per km^2^ and a minimum population of 5,000. NUTS 3 regions are classified as follows on the basis of the share of their population in rural areas (Eurostat, 2021):

- *Predominantly rural if the share of the population living in rural areas is higher than 50%*,
- *Intermediate if the share of the population living in rural areas is between 20% and 50%*,
- *Predominantly urban if the share of the population living in rural areas is below 20%.*

To resolve the distortion created by extremely small NUTS 3 regions, for classification purposes regions smaller than 500 km^2^ are combined with one or more of their neighbors. The size of the urban centers in the region is considered. A predominantly rural region that contains an urban center of more than 200,000 inhabitants making up at least 25% of the regional population, becomes intermediate. An intermediate region that includes an urban center of more than 500,000 inhabitants making up at least 25% of the regional population becomes predominantly urban. In the NUTS codes of Czechia, the three levels are (Table 1):

**TABLE 1:** NUTS statistical regions of Czechia.

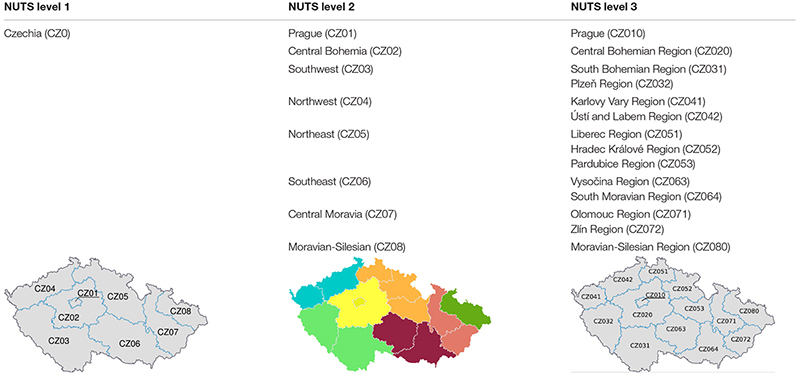

**Own elaboration, 2021.**

It is essential to highlight those rural statistics are territorial in nature, in contrast to sectoral statistics that focus on a single activity. People in rural areas are typically engaged in several different economic activities beyond agriculture, fisheries, and forestry, for example, mining and quarrying and craft production. Some of the main challenges facing rural areas include malnutrition, food insecurity, poverty, limited adequate health and education services, a lack of access to other basic infrastructure, and the under-utilization of labor. In formulating a rural development policy, the FAO draws on issues identified in the 2030 Agenda for Sustainable Development while acknowledging that rural areas have particular characteristics that present unique challenges. These include, among others: the dispersion of rural populations; topographical features (terrain and landscapes) that may act as a barrier for the efficient provision of infrastructure; an (over) reliance on the agricultural sector; ensuring that natural resources and environmental quality are protected (Dijkstra et al., 2021).

We can state that rural tourism is tourism which takes place in non-urbanized areas. These areas typically include (but are not limited to) national parks, forests, countryside areas, and mountain areas.

**FIGURE 1:**
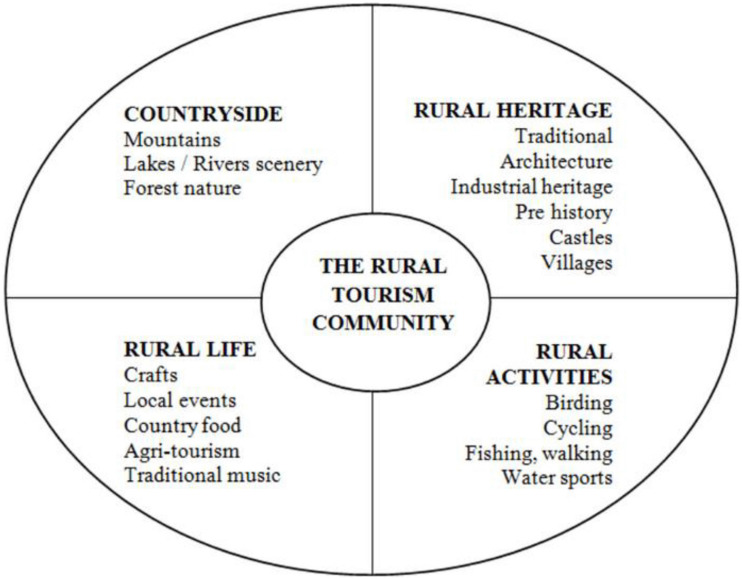
Effects of rural tourism (Nulty, 2004).

Rural tourism is closely aligned with the concept of sustainable tourism, given that it is essentially linked to green spaces and commonly environmentally friendly forms of tourism, such as hiking or camping. Lane (2009) states that pure rural tourism is defined as a tourism type located in rural areas. Rural tourism has a different scale, character, and function (Sharpley and Roberts, 2004). Šimková (2007) states that rural tourism or agro-tourism becomes very popular, especially in economically developed countries. Its economic and socially positive impact allows farmers to gain additional financial sources and create new job positions for other local people. It is a very positive and ecological form of tourism. Unlike uncontrolled, mass, and purely commercial tourism, these leisure activities negatively impact the environment. Nulty (2004) claims that the rural tourism industry interlinks with a range of activity types, thus bringing economic benefit to various areas (Figure 1).

In rural areas, there are positive as well as negative impacts (White, 1985; Oppermann, 1995; Sharpley and Sharpley, 1997; Butler and Hall, 1998; Hall, 1999; Roberts and Hall, 2001; Sharpley and Roberts, 2004; Lane, 2009; Dashper, 2014; Nair et al., 2015; Dewi and Djunaid, 2019):

- *Positive social-economic impacts*: provide source of new, alternative, or supplementary income and employment, help reduce gender and other social power imbalances, encourage collective community activity, provide opportunities for retaining population in areas that might otherwise experience depopulation, enable areas to be repopulated and overall multiplier effects, although in rural areas these tend to be lower.
- *Negative social-economic impacts*: economic leakages, local price inflation, labor in-migration, distort local employment structure, distort local housing market, reinforce perception of women’s employment as low paid and part-time and an extension of “the domestic role,” self-contained complexes with tenuous links to the local economy, and seasonal patterns of demand.
- *Positive cultural impacts*: reinvigorate local culture, instill sense of local pride, self-esteem, and identity.
- *Negative cultural impacts*: manufacture of distort local culture for commodification and staged authenticity, destroy indigenous culture.
- *Positive physical (built and “natural”) impacts*: contribution to conservation and protection, assist refurbishment and re-use of abandoned properties.
- *Negative physical (built and “natural”) impacts*: habitat destruction, littering, emissions and other forms of pollution, congestion, and new construction sprawl, perhaps grafted on to existing settlements.

Actually, the tourism industry is facing a new challenge, but, despite the catastrophic effects caused by the COVID-19 pandemic, it is evident that when the projects work at their maximum capacity, they will support all stakeholders’ economies (Li et al., 2021). Overtourism is a problem addressed by many researchers at many levels. It is a phenomenon that is inconspicuous and slow. Diaz-Parra and Jover (2021) state that overtourism illustrates how the residents lose the right to everyday life in their local spaces. Mechanisms of commodification and gentrification contribute to socio-spatial injustices and also make some places “inauthentic” for visitors and residents. Therefore, most destinations can only start to deal with it late. Overtourism is even worse by the recommendations of celebrities or influencers or the making of popular movies in the area (Závodná, 2020). Overtourism has become a significant concern for an increasing number of destinations as tourism numbers continue to grow, stimulated by general economic and technological growth and the expansion of the global middle Class (Rončák, 2020). Overtourism is an issue that arises inconspicuously and gradually. Therefore, most destinations start to solve it only when there are too many difficulties, and sometimes it is too late to maintain the original state of the destination. While visitors want to experience the locals’ authentic feeling, the real locals are moving to places where they are not threatened by excessive night noise, light, and crowds of entertaining people. However, it is not only local people who are suffering. The same negative impact is also experienced by infrastructure, monuments, or nature (Závodná, 2020).

The attractiveness of tourism resources has several barriers in the long-term considering the community. The challenges may include financial issues, land ownership, or problems of community participation and involvement. Without creating opportunities for residents to participate in the decision-making process around benefits generated from the tourism development, the decision is more complicated. According to this challenge, some community members are operating against stronger competitors and poor market conditions (Lo and Janta, 2020).

It is not straightforward to evaluate the attitudes of local inhabitants to tourism development. An outstanding illustration of the change in residents’ attitude toward tourists is the Doxey index (Doxey, 1975). The appearance of negative attitudes among residents toward tourists results from the social carrying capacity limits being exceeded, the inability to accept changes as they occur (Szromek et al., 2020). Doxey irritation index (Irridex) is based on the understanding of residents’ attitude change toward tourists and tourism development in different stages of a destination’s life cycle. This model assumes the resulting circumstances with negative sociocultural impacts can lead to irritation in the local community. Its four stages of euphoria, apathy, irritation, and antagonism explain the residents’ deteriorating responses to tourism development Table 2).

**TABLE 2:** Doxey irritation index.

Stages of irritation	Characteristics
Euphoria	- Small number of tourists - Local community welcomes tourists - Local people are enthusiastic and thrilled by tourism development - Missing developing plan - New opportunities for local people
Apathy	- Increasing number of tourists - Relationship between tourists and residents becomes formalized - Tourists are taken for granted - Tourism profit perception for a destination
Irritation	- Significant growth of tourists - It is impossible (or very difficult) to handle number of tourists without expansion of facilities - The industry is near the saturation point - Tourist oriented services - Socio-pathological phenomenon (criminality, delinquency)
Antagonism	- Very high number of tourists (overtourism) - Hostility and resistance to tourists - Especially negative impacts are perceived - Finger pointing tourists (tourists are seen as the harbinger of all that is bad) - Lose of destination authenticity

**Own elaboration according to Pavlić and Portolan (2015); Szromek et al. (2020).**

**FIGURE 2:**
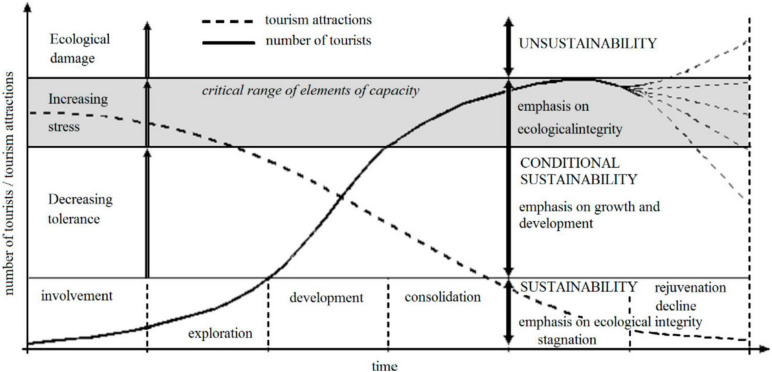
Sustainable Development and Tourism Area Life Cycle. Szromek et al. (2020).

During the first stage, the number of tourists is small and the local community welcomes tourism. In the apathy phase, the number of tourists increases, and the relationship between tourists and residents becomes formalized. Irritation is the phase when residents become concerned about tourism due to the significant growth of arrivals and increasing competition for resources. In the last stage, antagonism, tourists become responsible for everything wrong that has happened in the host community (Pavlić and Portolan, 2015). Irridex as a concept is not based on any detailed empirical research (Fridgen, 1991). The model assumes a degree of homogeneity and positive linear relationship and ignores complexities within the host community as well as the multidimensionality of tourism impacts (Cordero, 2008). Pavlić and Portolan (2015) state that the Irridex is a theoretical model that requires constant and contextual empirical tests, as it can be changed depending on geographical locations, problems, and even aims of a study. Nonetheless, the model serves as a valuable framework for the understanding of the changing resident attitudes and developmental stages of a destination (Figure 2).

## Materials and Methods

The goal of this manuscript is to analyze the attitudes of local residents to the development of tourism in the urban monument zone Předhradí. In connection with the research goal, the following research questions were posed: What are the significant negative impacts of tourism development according to locals? Do locals see positive impacts on their quality of life conducted with tourism development?

Primary data were collected in a questionnaire survey for residents who have a permanent residence in a municipality of Předhradí in 2020. The questionnaire consisted of thirteen questions which directly concerned the municipality of Předhradí. These were open, semi-open and closed questions. It was a random selection. Because it is not easy to organize empirical research, the administrators tried to behave in a user-friendly way. All the participants were informed about the research and anonymity of the questionnaire. The participants were willing to participate in the research and also had the opportunity to contact the interviewer via the email stated on the questionnaire list and inform themselves about the research results.

Based on the demographic distribution of the population of the municipality of Předhradí determined through the Czech Statistical Office, a percentage of the sample was planned. The municipality of Předhradí has a total of 417 inhabitants, of which 205 women (49.16%) and 212 men (50.84%). The examined sample consisted of 107 respondents of different age categories (Table 3), of which 52 were women (48.60%) and 55 men (51.40%), so it can be stated that this is an even distribution. The effort in compiling the surveyed sample was to get as close as possible to the structure of the basic sample.

**TABLE 3:** Structure of respondents.

		N	%
Gender	Female	52	48.60
	Male	55	51.40
	Total	107	100.00
Age	18–24	13	12.15
	25–34	14	13.08
	35–44	20	18.69
	45–54	18	16.82
	55–64	19	17.76
	65 and more	23	21.50
	Total	107	100.00
Length living in the municipality	To 5 years	10	9.34
	6–19 years	34	31.78
	20–40 years	30	28.04
	41 years and more	33	30.84
	Total	107	100.00

**Own elaboration, 2021.**

Our results come from quantitative research methods. In this research, we used mathematical, statistical methods, the analysis method—also Correspondence Analysis (CA) and the method of generalization. For evaluating the results, the Statistica 13 EN Program was used. Using the graphic tools of this CA, it is possible to describe an association of nominal or ordinal variables and to obtain a graphic representation of a relationship in multidimensional space—for the readers; it is easier to understand. The analysis provides further evidence that correlations exist between variables.

Correspondence analysis is a multivariate statistical technique. It is conceptually similar to principal component analysis but applies to categorical rather than continuous data. In a similar manner to principal component analysis, it provides a means of displaying or summarizing a set of data in a two-dimensional graphical form (Zámková and Prokop, 2014).

All data should be non-negative and on the same scale for CA to be applicable, and the method treats rows and columns equivalently. It is traditionally applied to contingency tables. CA decomposes the chi-squared statistic associated with this table into orthogonal factors. The distance between single points is defined as a chi-squared distance. The distance between the i-th and i′-th row is given by the formula

$$\text{D}\,\left(i,{\text{i}}^{\prime }\right)=\sqrt{\sum_{j=1}^{c}\frac{{\left({\text{r}}_{i\,j}-{\text{r}}_{i^{\prime }\,j}\right)}^{2}}{{\text{c}}_{j}}}$$

where r_*ij*_ are the elements of row profiles matrix R and weights c_*j*_ correspond to the elements of column loadings vector cT, which is equal to the mean column profile (centroid) of the column profiles in multidimensional space. The distance between columns j and j′ is defined similarly, weights correspond to the elements of the row loadings vector r and sum over all rows. In correspondence analysis we observe the relation between single categories of two categorical variables. The result of this analysis is the correspondence map introducing the axes of the reduced coordinates system, where single categories of both variables are displayed in graphic form. The aim of this analysis is to reduce the multidimensional space of row and column profiles and to save as far as possible original data information. Each row and column of the correspondence table can be displayed in c-dimensional (r-dimensional, respectively) space with coordinates equal to the values of the corresponding profiles. The row and column coordinates on each axis are scaled to have inertias equal to the principal inertia along that axis: these are the principal row and column coordinates (Hebák et al., 2007).

For the correspondence analysis model, the degree of dispersion of points is defined, i.e., row and column categories, the so-called total inertia. The term inertia comes from mechanics, where it is defined as the sum of the product of mass and square distances from the centroid of all the object’s particles. Geometrically, inertia expresses the degree of dispersion of points in multidimensional space and it can be understood as an analogy to the dispersion known from statistical modeling. In a correspondence analysis, the total inertia (I) is equal to the weighted average (with weights p_*i*__++_) chi-square of the distance of the row profiles from their average/mean (vector c), (Greenacre, 2007; Hebák et al., 2007; Beh and Lombardo, 2014):

$$\text{I}=\sum_{i=1}^{r}{\text{p}}_{i+}\,{\left({\text{r}}_{i}-\text{c}\right)}^{T}\,{\text{D}}_{c}^{-1}\,\left({\text{r}}_{i}-\text{c}\right)$$

The same as the weighted average (with weights p_+__*j*_) chi-square of the distance of the column profiles from their average (vector r)

$$I=\sum_{j=1}^{c}p_{+j}\,{\left(c_{j}-r\right)}^{T}\,D_{r}^{-1}\,\left(c_{j}-c\right)$$

A significant part of the total inertia of the original table is usually explained by the first several axes. That is why it is generally sufficient for the result of the correspondence analysis to be represented in the space of the first two or three ordinal axes. Total inertia equals the sum of all eigenvalues of the matrix. Therefore, it is possible to specify how many ordinal axes it is reasonable to interpret. This can be decided in either of two ways. (1) We set a threshold value (e.g., 80%) and determine how many axes have a cumulative inertia higher than the set threshold value. (2) We interpret the ordinal axes whose eigenvalue is above-average, i.e., higher than the average of all eigenvalues.

The contributions of the row points to the inertia in the corresponding dimension are defined by the quotient

$$\frac{r_{i}\,f^{2}\,i\,k}{\lambda \,\left(k\right)}$$

where ∫_*ik*_ corresponds with the elements of the matrix F (the score of the i-th row category in the k-th dimension), r_*i*_ elements of the row loadings vector and ^λ^ (k)is inertia expressed by the k-th dimension (an eigenvalue of the matrix). A contribution of the row points to inertia expresses the relative degree of the effect of the given category on the final orientation of the main axes.

In a similar fashion, the contributions of column points to inertia are expressed in the corresponding dimension

$$\frac{c_{j}\,g^{2}\,j\,k}{\lambda \,\left(k\right)}$$

For each row category, we can calculate the total row inertia, defined as

$$\sum_{n=1}{\text{r}}_{j}\,{\text{f}}^{2}\,\text{j}\,k$$

Similarly, for column categories, the total column inertia is defined as

$$\sum_{k}{\text{c}}_{j}\,{\text{g}}^{2}\,\text{j}\,k$$

The values of inertia for individual columns and rows give us an indication of the significance of the various categories on the resulting ordination (Greenacre, 2007; Hebák et al., 2007; Beh and Lombardo, 2014).

## Results and Discussion

Tourism development brings a lot of positive impacts for destinations, e.g., economic growth, new working possibilities, new business opportunities, etc. It stimulates the local economy. The effects of tourism development are not only positive. Also, few negative influences can be observed. The most controversial is the number of tourists (Yang et al., 2020). As the prosperity of the world increases, more people are able to enjoy trips that were once only possible for the select few. Dream destinations have now become possible for many throughout the world. This has led to too many people trying to visit the “must-see” locations of the world creating “tourist ghettos.” This inevitably leads to disappointment as people are reduced to a melancholy mass (Pompurová et al., 2020). The positive relationships between tourism psychological empowerment, tourism economic empowerment, and participation willingness would be more robust when village residents’ participation ability is higher (Yang et al., 2020).

Our research was focused on the attitudes of locals to tourism development in the municipality of Předhradí. We conducted research on 107 persons, which is a quarter of all inhabitants.

**FIGURE 3:**
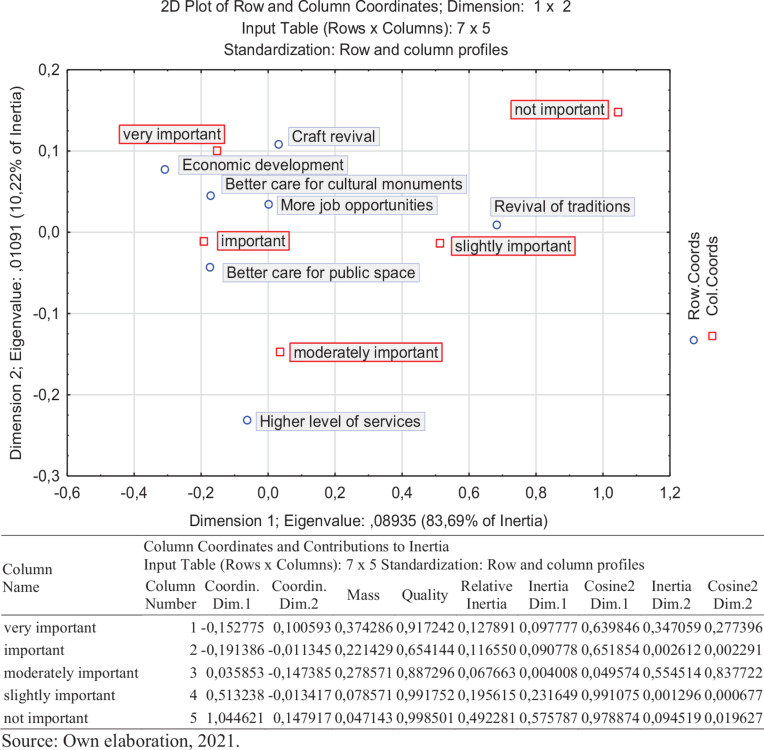
Positive impacts of tourism development in the municipality of Předhradí. Own elaboration, 2021.

The most visible positive impacts for them were economic development in the local area, more job opportunities, and better care for cultural monuments. So, we can state that locals consider the economic benefits as the most critical impacts of tourism development. As the less significant impact, local people see the revival of local traditions (Figure 3).

**FIGURE 4:**
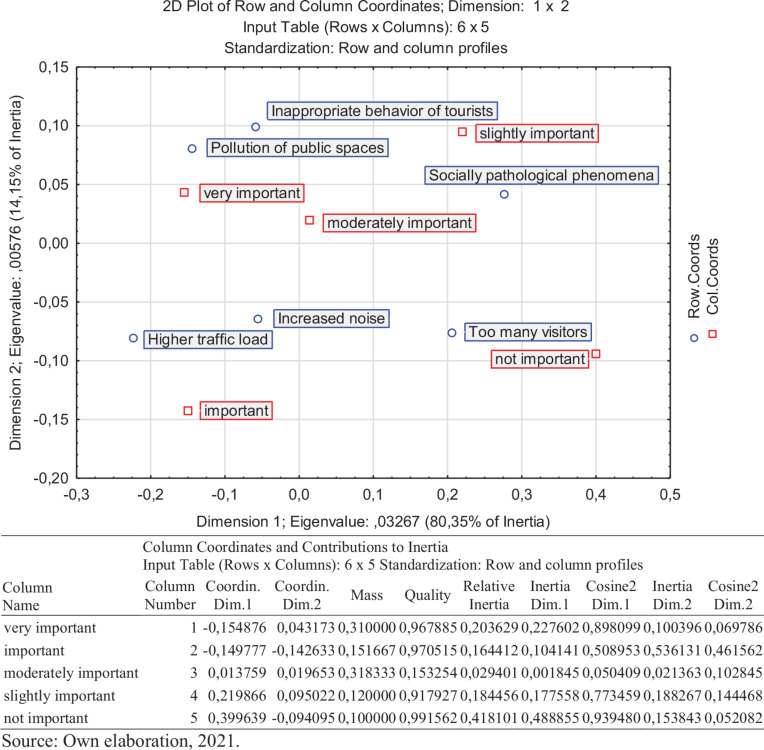
Negative impacts of tourism development in the municipality of Předhradí. Own elaboration, 2021.

From negative impacts were the pollution of public spaces and the inappropriate behavior of tourists marked as the biggest threat of tourism development for the local area. We assumed as most negative impacts higher traffic load or increased noise. Local respondents perceived these impacts as necessary. They do not see them as a problem. The reason should be in inexperience of local people with tourists and tourism development. Up to 59% of surveyed locals perceive the future influx of tourists as a positive state. Only 5% of surveyed locals do not agree with the castle Rychmburk disclosure. On the other hand, more than two-thirds of interviewed locals (67%) see it as a positive act (Figure 4).

For tourism development in the rural area is critical participation of the local community. Locals should know the positive impacts of tourism. They should participate and support tourism development. But they also have to feel the positive effects in their life or business. The negative attitudes of local people should be discussed with the local autonomy and solutions should be found. Resident support is crucial to tourism development’s sustainability by focusing on the mixed relationship between (a) residents’ perceived personal benefits from tourism and perceived negative tourism impacts and (b) perceived negative tourism impacts and residents’ support for tourism. Tolerance for tourism development revealed by residents implies that higher tolerance reduces the effect of perceived negative impacts on residents’ support for tourism development (Qin et al., 2021).

**FIGURE 5:**
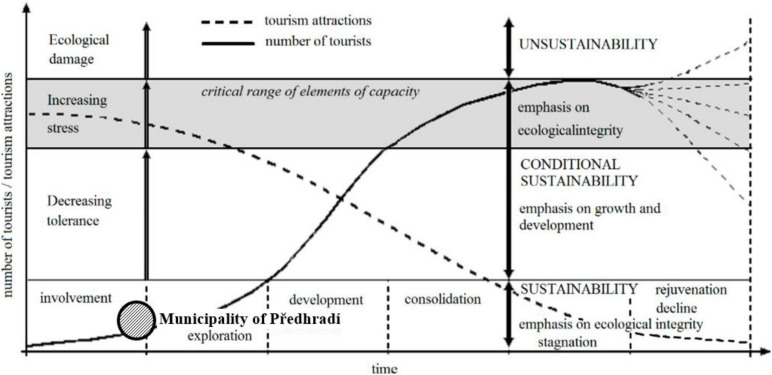
Identification of Municipality Předhradí in the Tourism Area Life Cycle (1). Own elaboration according to Szromek et al. (2020).

**FIGURE 6:**
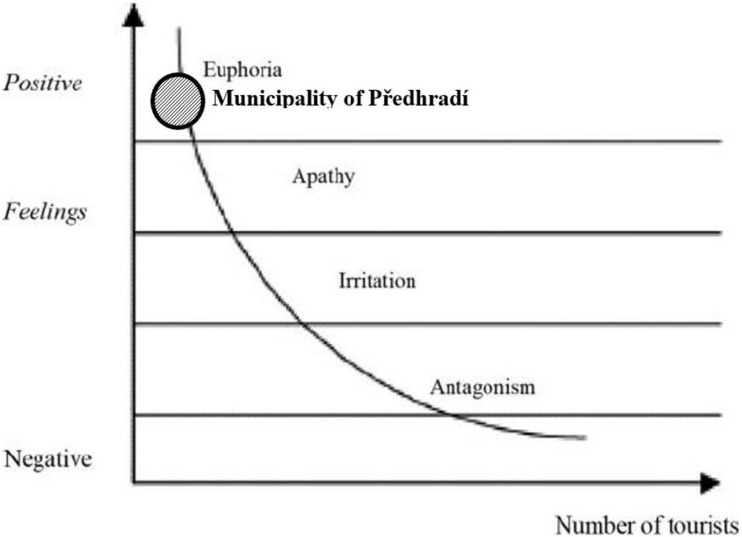
Identification of Municipality Předhradí in the Tourism Area Life Cycle (2). Own elaboration according to Babu and Munjal (2015).

Currently, the Rychmurk castle is not accessible to the public. In the castle, there is situated the home for people with mental illness. Tourists can visit the castle during opening hours or on request. They also can see the educational route around the castle. The local government wants to open the castle for the public and tourists in a more extensive range. In the municipality in Předhradí are located two restaurants. There is no official accommodation possibility. Tourists can use accommodation facilities in the town of Chrudim (district town), which is 30 km far. The municipality of Předhradí belongs to Chrudim administrative district. In Chrudim are located fourteen accommodation facilities. The nearest town with accommodation facilities to Předhradí is the town of Skuteč (4 km far). There are located three accommodation facilities, only two of them are open during the whole year. The Chrudim administrative area was visited by 105 thousand visitors in 2019. It is around one-fifth of all visitors in the Pardubice region. According to research results, we classified the municipality of Předhradí to the stage of irritation “euphoria.” Ironically, being commodified, marked, and used in public policy focused on maximizing economic output implies an essential ideological component. It is a new phenomenon with consequences in the tourism campaigns selling authentic happiness (Phillips et al., 2021). The locals have only some pieces of information about tourism development, and the developing plan is missing. A small number of tourists actually visit the area. The majority of them are 1-day visitors or excursionists. Locals have open attitudes toward tourists and welcome tourists. The majority of local inhabitants see especially positive impacts of tourism development. They assume economic development and increasing job opportunities (around 60% of respondents). Tourism development is also seen as a toll of cultural heritage revitalization. A business model transformation based on cultural heritage revitalization is most frequently occurring, thanks to touristic ventures aiming at production or extraction facilities. Some of these places appear with new touristic functions, even if initially, they have been designed for other purposes. Such transformed business models effectively preserve and save cultural heritage from degradation (Szromek et al., 2020). The biggest concerns evoke the pollution of public spaces and the inappropriate behavior of tourists. Local inhabitants do not see the potentially high number of tourists, increasing noise, or criminality as a problem. We guess that these aspects of tourism development are not highlighted due to local people’s inexperience with their tourism popularity (Figures 5, 6).

## Limitations and Directions for Future Research

This study focuses on the evidence from Czechia and considers that the local population’s attitudes may change from different countries. It represents a limitation that opens an opportunity for future research. When the tourism development component is in the research, the effect of brand equity can influence the results. We suggest extending the analysis to other sensitives effects of tourism development through the local population’s attitudes in different countries (Passagem et al., 2020).

Clearly, the impact of the COVID-19 pandemic has had a severe impact on the tourism and hospitality businesses throughout the world, and in Czechia is expected the same. To contribute to business longevity, we suggest future research to explore the growth and decline of business organizations within government policy dynamics to support those organizations. Probably, social attitudes have changed, and these have primarily reduced the ability of the environment to keep the pre-COVID-19 pandemic levels of business. Hospitality businesses need to change and innovate to survive.

Although some municipalities, e.g., Předhradí, revealed some “euphoria,” most local inhabitants confirm positive impacts of tourism development. We must consider that economic growth seems the channel that supports human development expansion. Tourism specialization’s potential impact on private and public incomes is the relevant developmental channel in transition economies (Croes et al., 2021).

We suggest applying a similar study after the pandemic crisis to compare the results and take some measures to adjust to the new reality. This kind of comparative study will be appreciated not only from the academic perspective as from a practical standpoint. For many reasons, Czechia can be a great example to reply to other geographical regions.

According to other findings, the COVID-19 pandemic as presenting opportunities for strategic innovation. The use of technology, particularly social media, to create innovative approaches to marketing and new delivery modes is one of the options and directions for future research. On the other hand, the direct personal contact that enables non-contact delivery provision can represent an opportunity for the future (Hemmington and Neill, 2021).

The actual crisis requires a more enlightened understanding to determine appropriate management strategies to promote sustainable behaviors. In another way, the residents’ behavior can profoundly influence the community (Ramkissoon, 2020).

## Conclusion

The goal of this manuscript was to analyze the attitudes of local residents to the development of tourism in the urban monument zone Předhradí. In connection with the research goal, the following research questions were posed: What are the significant negative impacts of tourism development according to locals? Do locals see positive impacts on their quality of life conducted with tourism development?

Considering the theoretical framework on attitudes of the local population, we found some relevant conclusions when we take evidence from Czechia. For example, in the Municipality Předhradí, the most significant concerns evoke public spaces’ pollution and tourists’ inappropriate behavior. If we consider the trends around the globe and the same in Czechia to protect the environment, there will probably be many changes in these subjects in the following years. Furthermore, the pandemic crisis appears with a negative impact and some transformational trends in the pollution levels.

On the other hand, the tourism development impacts can occur positively or negatively, so the social-economic effect on the local population can process consequences, as evidenced in our case study. We assumed that local respondents perceived some negative impacts as necessary to increase the economic perspective, as they referred to in the higher traffic load or increased noise.

Our study’s highlight positive impact was economic development in the local area, with better care for cultural monuments and more job opportunities. According to the new challenges presented by the COVID-19 pandemic, our study must be considered in further research to compare the results and adjust the new reality measures.

Locals are an essential part of the destination. Together with businesses and tourist attractions, they form the destination’s climate; locals, business people, and visitors are interconnected and influence each other. Information and cooperation of residents are important for the development of tourism in the area. It is necessary to treat them with respect, taking into account their worries and ideas. This behavior model makes it possible to reduce the negative social impact of tourism development on the local community. If local people have the opportunity to participate in decisions about the direction of development in the area, attention is paid to their views, concerns, and suggestions for improvement; it has positive effects. Thus, residents will rather form tourism destinations in cooperation with local governments and entrepreneurs.

Furthermore, it is possible to reduce the level of criticism from local people in the event of negative tourism development manifestations. They will be more tolerant of tourists and look for standard ways of developing tourism in a destination that suits the majority. Local people should also have the opportunity to actively participate in the development of tourism, e.g., in the form of discussions on development plans, participation in revitalizing the environment, reviving local traditions, promoting local accommodation, etc. They should see the positive effects of tourism development in their daily lives. A simple saying works “satisfied locals generate satisfied tourists.”

## Data Availability Statement

The raw data supporting the conclusions of this article will be made available by the authors, without undue reservation.

## Ethics Statement

The study involving human participants was reviewed and approved by the Ethics committee at the College of Polytechnics Jihlava. The participants provided their written informed consent to participate in this study.

## Author Contributions

IL: topic proposed. IL and PS: experimental design and data collection. IL, PS, and NA: manuscript writing. PS and NA: content proofreading. All authors contributed to the article and approved the submitted version.

## Conflict of Interest

The authors declare that the research was conducted in the absence of any commercial or financial relationships that could be construed as a potential conflict of interest.
